# Modulation of sphingosine receptors influences circadian pattern of cardiac autonomic regulation

**DOI:** 10.14814/phy2.12870

**Published:** 2016-09-13

**Authors:** Sakari Simula, Tomi P. Laitinen, Tiina M. Laitinen, Päivi Hartikainen, Juha E. K. Hartikainen

**Affiliations:** ^1^ Department of Neurology Mikkeli Central Hospital Mikkeli Finland; ^2^ Department of Clinical Physiology and Nuclear Medicine Kuopio University Hospital and University of Eastern Finland Kuopio Finland; ^3^ Neuro Center Department of Neurology Kuopio University Hospital and University of Eastern Finland Kuopio Finland; ^4^ Heart Center Kuopio University Hospital and University of Eastern Finland Kuopio Finland

**Keywords:** Autonomic nervous system, circadian rhythm, multiple sclerosis, sphingosine

## Abstract

Fingolimod is an oral sphingosine‐1‐phospate (S1P) receptor modulator for the treatment of relapsing‐remitting multiple sclerosis (RRMS). In addition to therapeutic effects on lymphoid and neural tissue, fingolimod influences cardiovascular system by specific S1P‐receptor modulation. The effects of S1P‐receptor modulation on the endogenous circadian pattern of cardiac autonomic regulation (CAR), however, are not known. We examined the effects of fingolimod on the circadian pattern of CAR. Ambulatory 24‐h ECG recordings were undertaken in 27 RRMS patients before fingolimod (baseline), at the day of fingolimod initiation (1D) and after 3 months of fingolimod treatment (3M). The mean time between two consecutive R‐peaks (RR‐interval) and mean values for measures of heart rate variability (HRV) in time‐ and frequency domain were calculated from ECG recording at daytime and nighttime. The mean night:day‐ratio of RR‐interval was 1.23 ± 0.12 at baseline, decreased temporarily at 1D (1.16 ± 0.12; *P *<* *0.01) and was higher at 3M (1.32 ± 0.11; *P *<* *0.001) than at baseline. The night:day‐ratio of HRV parameters reflecting parasympathetic cardiac regulation (pNN50, rMSSD, HFnu) decreased at 1D but recovered back to baseline at 3M (*P *<* *0.05 for all). On the other hand, the night:day‐ratio of TP, a parameter reflecting overall HRV gradually decreased and was lower at 3M than at baseline (*P *<* *0.05). Our findings suggest that physiological relation between the circadian pattern of RR‐interval and overall HRV as well as parasympathetic cardiac regulation becomes uncoupled during fingolimod treatment. In addition, fingolimod shifts the circadian equilibrium of CAR toward greater daytime dominance of overall HRV. Accordingly, S1P‐receptor modulation influences circadian pattern of CAR.

## Introduction

Fingolimod is a sphingosine analogue used for the treatment of relapsing‐remitting multiple sclerosis (RRMS) (Cohen et al. 2010; Kappos et al. 2010; Calabresi et al. 2014). The disease‐modifying effects of fingolimod on RRMS are mediated via sphingosine‐1‐phosphate (S1P)–receptor modulation on lymphoid and neural tissue (Matloubian et al. 2004; Massberg and von Andrian 2006; Brinkmann et al. 2010; Chun and Hartung 2010) , but S1P‐receptors are also present in the cardiovascular system (Mizugishi et al. 2005).

The initial vagomimetic cardiovascular effects of fingolimod result from S1P1‐receptor agonism, which is G‐protein coupled similar to acetylcholine receptors (Koyrakh et al. 2005). The vagomimetic effects of fingolimod attenuate gradually due to downregulation of S1P1‐receptors. Consequently, the S1P‐receptor profile becomes dominated by S1P2 and S1P3 receptors on the cell surface (Camm et al. 2014). However, fingolimod itself does not bind to S1P2‐receptors, and its affinity for the S1P3‐receptor is considerably weaker than that of physiologically secreted (endogenous) sphingosine (Brinkmann et al. 2002; Mandala et al. 2002).

In healthy subjects, cardiac autonomic regulation has an intrinsic circadian pattern with parasympathetic dominance at nighttime (Huikuri et al. 1994; Li et al. 2011). Impairment of the physiological circadian pattern is associated with an increased risk of cardiac events such as myocardial ischemia, myocardial infarction, malignant arrhythmias, and sudden cardiac death peaking in the early morning (Muller et al. 1989; Bernardi et al. 1992; Huikuri et al. 1992; Carney et al. 2014). However, all the mechanisms responsible for the circadian pattern of cardiac autonomic regulation are not known.

Cardiac autonomic regulation can be assessed by heart rate variability (HRV). Acquisition of continuous electrocardiogram (ECG) by ambulatory 24‐h recording allows identification of nighttime and daytime signals and thus, circadian pattern in cardiac autonomic regulation can be obtained.

In this prospective study, we evaluated the effect of fingolimod on the circadian pattern of cardiac autonomic regulation in RRMS patients. Cardiac autonomic regulation was assessed from HRV at night and by day before fingolimod initiation, during the first 24‐h after fingolimod initiation and after 3 months of continuous fingolimod treatment.

## Methods

### Ethical approval

Before participating the study, each patient gave a written informed consent after full explanation of the purpose, nature, and the risks related to the procedures used. The ethics committee of Kuopio University Hospital approved the study protocol and the research was carried out in accordance with the Declaration of Helsinki (2008) of the World Medical Association. The study was registered at ClinicalTrials.gov (NCT01704183).

### Study protocol

The patients underwent 24‐h ambulatory ECG recording 20 ± 16 days before fingolimod treatment for baseline (B), at the day of fingolimod initiation (1D), and after 3 months of (88 ± 7 days) fingolimod treatment (3M). The ECG recording at 1D was started prior to fingolimod initiation before 10:00 am and also well before noon for baseline and 3M recordings. The mean values of the different HRV parameters were calculated for the nighttime (midnight to 6 am) and daytime (first 12 post‐dose hours at 1D and first 12 h of the recording at B and 3M). The neurological disability related to RRMS was assessed by Expanded Disability Status Scale (EDSS) for each patient at baseline.

### Patients

This study consisted of 27 RRMS patients (16 women and 11 men) who were eligible for the disease‐modifying fingolimod treatment on a clinical basis. Fingolimod was prescribed as a second‐line immunomodulative treatment for RRMS either due to lack of effect or significant side effects of first‐line treatments. All patients were followed at least 6 h after the first dose of fingolimod to overcome the nadir heart rate before hospital discharge, as required by drug label. No patients needed overnight observation in the hospital.

The patients were 43 ± 11 years of age and the diagnosis of RRMS was set 10 ± 7 years before the study. The median value of EDSS was 3.5 (range: 1.0–6.5) at baseline. None of the patients demonstrated clinical disease activity in RRMS during the study. Five patients (19%) had one or more of the following co‐morbidities: three patients (11%) were adequately treated with hormonal substitution for hypothyroidism, two patients (7%) had type‐1 diabetes mellitus with insulin‐treatment, one patient (4%) had asthma, and one patient (4%) had optimally treated hypertension combined with Raynaud phenomenon. No changes in medication other than the initiation of fingolimod were allowed during the study. Previous immunomodulatory treatment for RRMS was discontinued at least 2 months before fingolimod initiation if shifted from natalizumab and at least a day before if shifted from interferon‐1b or glatiramer acetate.

### Acquisition of ECG signal

Twenty‐four‐hour electrocardiogram (ECG) was acquired by ambulatory Schiller Medilog AR12plus recorders (Schiller Medilog, Schiller AG, Switzerland) with a sampling frequency of 250 Hz. Three bipolar ECG leads (modified chest leads V1 and V5 and modified aVF) were used.

Digital ECG recordings were read to Darwin Holter analysis system (Schiller Medilog, Schiller AG, Switzerland) and they were exported in MIT‐format for the further analyses. Preprocessing of RR‐intervals was performed with an automatic R‐peak identification algorithm. Correct identification was manually confirmed. Manual adjustment of R‐peak localization was applied when needed. Cardiac extrasystoles occurred rarely and were adjusted by interpolation. The time window for HRV analysis was 1 h and was equal in all patients. Normal daily living was allowed during ambulatory ECG recordings including the 6 h in‐hospital observation at the day of fingolimod initiation.

### Analysis of heart rate variability

The values for different HRV parameters in time domain and frequency domain were calculated according to the recommendations (Task Force of the European Society of Cardiology and the North American Society of Pacing and Electrophysiology 1996) and using the WinCPRS software (Absolute Aliens Oy, Turku Finland). The standard deviation of all RR‐intervals (SDNN), the percentage of normal RR‐interval with duration more than 50 msec different from the previous normal RR‐interval (pNN50), and the root mean square of successive differences in RR‐interval (rMSSD) were calculated to assess HRV in time domain. Frequency domain analysis of HRV was performed with fast Fourier transformation (linear detrending, resampling 5 Hz uneven, Hanning‐window). The total power (TP) spectrum of RR‐interval variability was divided into very low frequency (VLF) (0.005–0.04 Hz), low frequency (LF) (0.04–0.15 Hz), and high frequency (HF) (0.15–0.40 Hz) bands. The integrals under the power spectral density functions were measured as absolute units (equal to area under the curves for the spectral densities) and as normalized units by dividing the power of LF (LFnu) and HF (HFnu) components by TP, from which the <0.04 Hz spectral component had been subtracted and multiplying by 100. In addition, the ratio of the LF power band and HF power band (LF:HF‐ratio) was calculated.

### Physiological correlates of HRV

High HRV associates with higher cardiac parasympathetic regulation and low HRV indicates lower cardiac parasympathetic regulation in general (Task Force of the European Society of Cardiology and the North American Society of Pacing and Electrophysiology 1996). However, the physiological mechanisms and signaling routes underlying the various components of HRV differ. In the time domain, parasympathetic activation is typically reflected by increase in pNN50 and rMSSD variables of HRV. In the frequency domain, the LFnu component is considered to reflect mainly sympathetic and the HFnu component mainly parasympathetic cardiac autonomic regulation (Akselrod et al. 1981; Pomeranz et al. 1985). Correspondingly, the LF:HF‐ratio indicates the sympatho‐vagal balance of cardiac autonomic regulation (Task Force of the European Society of Cardiology and the North American Society of Pacing and Electrophysiology 1996).

### Statistical analyses

The normal distribution of values was assessed by Kolmogorov–Smirnov test. The values with non‐normal distribution were logarithmically (ln) transformed before any further statistical testing. The repeated measures ANOVA was applied to study differences between measurements at baseline, 1D and 3M. Thereafter, the significances of the differences between two specific time points were tested by paired sample t‐test. To test the significances of differences between two groups, independent samples *t*‐test were used for continuous variables. Results are expressed as mean ± standard deviation (SD), unless otherwise indicated. All analyses were conducted at the two‐tailed level and a *P*‐value <0.05 was considered statistically significant. The statistical analyses were performed using IBM SPSS statistics (version 19, 1989‐2010 SPSS Inc, Chicago).

## Results

**Figure 1:**
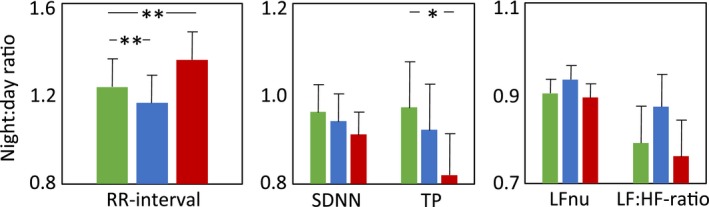
The average night to day ratio of RR‐interval (mean ± SEM), parameters reflecting overall heart rate variability (SDNN and TP), sympathetic cardiac regulation (LFnu) and sympatho‐vagal balance (LF:HF‐ratio) before fingolimod treatment (green), at the day of fingolimod initiation (blue), and after 3 months of treatment (red). The values are mean ± SEM;* n *=* *27. *P <* *0.05, **P <* *0.01.

The mean RR‐interval was longer at night than by day at baseline (958 ± 163 msec vs. 790 ± 129 msec; *P *<* *0.001), 1D (1034 ± 148 msec vs. 890 ± 112 msec; *P *<* *0.001), and 3M (1003 ± 159 msec vs. 763 ± 103 msec; *P *< 0.001), respectively. The average night to day ratio of RR‐interval was 1.23 ± 0.12 at baseline, decreased at 1D (1.16 ± 0.12; *P *<* *0.01) and was higher at 3M (1.32 ± 0.11; *P *<* *0.001) than at baseline (Fig. 1).

### Heart rate variability at night and during the day

The overall HRV assessed with SDNN in the time domain and with TP in the frequency domain did not differ between nighttime and daytime at baseline, 1D or 3M (Table 1).

**Table 1:** Heart rate variability during night and day before fingolimod initiation (B), at the day of fingolimod initiation (1D) and after 3 months of fingolimod treatment (3M)

	Night	Day	Significances
Time domain			
SDNN (msec)			
B	83 ± 28	89 ± 29	ns
1D	93 ± 28§§	103 ± 29§§§	ns
3M	67 ± 23§§§	74 ± 20§§§	ns
pNN50 (%)			
B	16 ± 13	9.2 ± 8.3	**
1D	23 ± 15§	16 ± 11§§	**
3M	10 ± 13§§§	4.7 ± 4.8§	*
rMSSD (msec)			
B	43 ± 30	31 ± 19	**
1D	51 ± 34§	40 ± 20§§	*
3M	29 ± 14§§§	22 ± 8§§	**
Frequency domain			
TP (msec^2^)			
B	5683 ± 3354	6402 ± 4128	ns
1D	6866 ± 3876§	8858 ± 4814§§	ns
3M	3405 ± 1768§§§	4635 ± 2588§§	ns
LFnu			
B	0.71 ± 0.16	0.78 ± 0.11	**
1D	0.67 ± 0.15§§	0.72 ± 0.10§§§	*
3M	0.69 ± 0.15	0.77 ± 0.09	**
HFnu			
B	0.28 ± 0.15	0.20 ± 0.10	**
1D	0.31 ± 0.14§	0.26 ± 0.09§§§	**
3M	0.30 ± 0.14	0.21 ± 0.08	**
LF:HF‐ratio			
B	4.37 ± 3.26	5.59 ± 2.87	**
1D	3.38 ± 2.75§§	3.76 ± 2.49§§§	ns
3M	3.49 ± 1.94	4.87 ± 248§	**

Significances: *P <* *0.05 and **P <* *0.01 in comparison between night and day. ^§^ *P *<* *0.05, ^§§^ *P *<* *0.01 and ^§§§^ *P *<* *0.001 in comparison to the value of corresponding parameter at baseline. Values are mean ± SD. *n *=* *27.

The parasympathetic components of HRV, such as pNN50 and rMSSD in the time domain and HFnu in the frequency domain were higher at night than by day at baseline, 1D as well as 3M (Table 1).

Mean LFnu, an index of cardiac sympathetic regulation, was lower at night than by day at baseline, 1D and 3M (Table 1). In addition, LF:HF‐ratio, a marker of sympatho‐vagal balance, was lower at night than by day at baseline (*P *<* *0.01) and 3M (*P *<* *0.01). However, there was no difference between the night and day LF:HF‐ratios at 1D (Table 1).

### Initial effects of fingolimod initiation on circadian pattern of heart rate variability

During the night, SDNN (*P *<* *0.01) and TP (*P *<* *0.05) as well as the parasympathetic components of HRV, such as pNN50 (*P *<* *0.05), rMSSD (*P *<* *0.05), and HFnu (*P *<* *0.01) were higher at 1D than at baseline (Table 1). On the other hand, LFnu (*P *<* *0.01) and LF:HF‐ratio (*P *<* *0.01) reflecting sympathetic cardiac regulation and sympatho‐vagal balance, respectively, were lower at 1D than at baseline during the night (Table 1).

During the day, SDNN (*P* < 0.001) and TP (*P *<* *0.01) as well as in pNN50 (*P *<* *0.01), rMSSD (*P *<* *0.01), and HFnu (*P *<* *0.001) were higher at 1D than at baseline (Table 1). On the contrary, LFnu (*P *<* *0.001) and LF:HF‐ratio (*P *<* *0.001) were lower at 1D than at baseline during the day (Table 1).

**Figure 2:**
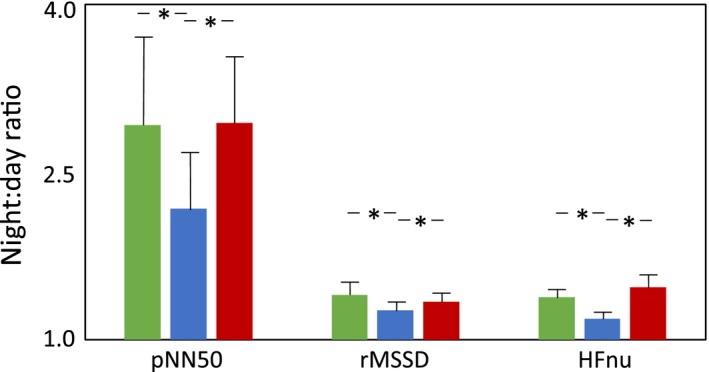
The average night to day ratio (mean ± SEM) of the heart rate variability parameters reflecting parasympathetic cardiac regulation before fingolimod treatment (green), at the day of fingolimod initiation (blue), and after 3 months of treatment (red). The values are mean ± SEM;* n *=* *27. *P <* *0.05.

The night to day ratio of parameters reflecting overall HRV (SDNN and TP) (Fig. 1, Table 2) as well as sympathetic cardiac regulation (LFnu) and sympatho‐vagal balance (LF:HF‐ratio) (Fig. 1, Table 2), remained unchanged from baseline to 1D. However, the night to day ratio of HRV parameters reflecting parasympathetic cardiac regulation, like pNN50 (*P *<* *0.05), rMSSD (*P *<* *0.05), and HFnu (*P *<* *0.05) were lower at 1D than at baseline (Fig. 2, Table 2).

**Table 2:** Night to day ratio for different heart rate variability measures before (B), at the day of fingolimod initiation (1D) and after 3 months of fingolimod treatment (3M)

	B	1D	3M	Significances B‐1D	Significances B‐3M
Time domain					
SDNN	0.96 ± 0.29	0.94 ± 0.29	0.91 ± 0.24	ns	0.09
pNN50	2.92 ± 3.88	2.17 ± 2.57	2.94 ± 2.85	*	ns
rMSSD	1.40 ± 0.55	1.26 ± 0.40	1.34 ± 0.45	*	ns
Frequency domain					
TP	0.97 ± 0.47	0.92 ± 0.53	0.82 ± 0.38	ns	*
LFnu	0.91 ± 0.16	0.93 ± 0.14	0.89 ± 0.17	ns	ns
HFnu	1.38 ± 0.49	1.19 ± 0.29	1.47 ± 0.59	*	ns
LF:HF‐ratio	0.79 ± 0.40	0.87 ± 0.42	0.76 ± 0.42	ns	ns

Significances: *P <* *0.05.

### Effects of 3 months of fingolimod treatment on circadian pattern of heart rate variability

After 3 months of fingolimod treatment, nighttime SDNN (*P *<* *0.001) and TP (*P *<* *0.001) as well as pNN50 (*P *<* *0.01) and rMSSD (*P *<* *0.001) were lower than at baseline (Table 1).

The daytime values of SDNN (*P *<* *0.001) and TP (*P *<* *0.01), as well as pNN50 (*P *<* *0.05), rMSSD (*P *<* *0.01), and LF:HF‐ratio (*P *<* *0.05) were lower at 3M as compared to baseline (Table 1).

Interestingly, a significantly lower night to day ratio was found in TP (*P *<* *0.05) at 3M than at baseline (Fig. 1, Table 2). A similar trend was found also in night to day ratio of SDNN at 3M (*P *=* *0.09) than at baseline (Fig. 1, Table 2). On the other hand, the night to day ratio of parameters reflecting parasympathetic cardiac regulation (pNN50, rMSSD, and HFnu) had recovered to baseline by 3 months of fingolimod therapy (Fig. 2, Table 2).

## Discussion

The new finding of our study was that fingolimod initiation influenced circadian pattern of cardiac autonomic regulation in patients with RRMS. The physiological nighttime dominance of cardiac parasympathetic regulation decreased at the day of fingolimod initiation. During continuous fingolimod treatment, however, the circadian pattern of parasympathetic components of HRV recovered, whereas the overall HRV demonstrated a gradual increase in day‐time dominance. The physiological coherence between circadian pattern of heart rate and HRV becomes uncoupled during fingolimod treatment.

Before fingolimod initiation, there was a clear circadian pattern of cardiac autonomic regulation in RRMS patients with parasympathetic dominance (high pNN50, rMSSD, HFnu, and low LFnu) at night. In addition, sympatho‐vagal balance (LF:HF‐ratio) demonstrated a circadian pattern with day‐time dominance. This is in line with previous studies reporting a circadian pattern of cardiac autonomic regulation in healthy subjects with parasympathetic dominance at night (Huikuri et al. 1994; Li et al. 2011).

Fingolimod initiation resulted in a change in the circadian pattern of cardiac autonomic control. The night to day ratio of the parasympathetic components of HRV decreased at the day of fingolimod initiation. The vagomimetic effects of fingolimod are most potent 4–5 h after initiation (Schmouder et al. 2011; Camm et al. 2014). In our patients, fingolimod was administered well before noon. Thus, the drug‐induced enhancement of the parasympathetic components of HRV was expected to be highest at daytime, that is, a few hours after the first dose of fingolimod. Accordingly, this resulted in enhancement of daytime parasympathetic cardiac regulation over the following night and a subsequent decrease in the night to day ratio of parasympathetic components of cardiac autonomic regulation.

After 3 months of fingolimod treatment, the circadian pattern of cardiac parasympathetic regulation had recovered completely. This is in line with an earlier experimental finding that pretreatment with fingolimod (resembling that of continuous fingolimod dosing) prevents the vagomimetic cardiac effects of sphingosine administration, but not restimulation with acetylcholine, which is the main neurotransmitter of the parasympathetic nervous system (Yamada 2002). Accordingly, the physiological circadian pattern of parasympathetic cardiac regulation in RRMS patients is present despite of the downregulation of S1P1‐receptors and subsequent alterations in S1P‐receptor profile induced by continuous fingolimod treatment.

Having demonstrated that the circadian pattern of parasympathetic cardiac regulation recovered during continuous fingolimod treatment, it was surprising to find that this was not the case for RR‐interval. Indeed, at 3 months, the night to day ratio of RR‐interval was higher than at baseline suggesting that the bradycardia related to fingolimod lasts longer at night than during the day.

Interestingly, the circadian pattern of overall HRV behaved differently from that of RR‐interval. The circadian pattern of TP showed increasing daytime dominance after 3 months of fingolimod treatment. A similar trend was seen also in SDNN, which is a surrogate marker for TP in time domain (Task Force of the European Society of Cardiology and the North American Society of Pacing and Electrophysiology 1996). Usually, overall HRV correlates with RR‐interval and it has been suggested that HRV could be substituted by RR‐interval in the assessment of cardiac autonomic regulation (Copie et al. 1996; Monfredi et al. 2014). Our study shows that this is not always the case. The circadian pattern of RR‐interval and overall HRV behaved oppositely. Night to day ratio of RR‐interval increased at 3 months, whereas that of TP and SDNN decreased compared to baseline.

Previously, we reported a decrease in overall HRV, suggesting increased sympathetic cardiac regulation, during 3 months of fingolimod treatment (Simula et al. 2016). According to our current findings, however, this seems to be asymmetric, as overall HRV (i.e., TP and SDNN) decreased more at night than during the day. This asymmetric shift may result from different interaction between endogenous cardiac autonomic regulation, sphingosine signaling, and fingolimod‐induced alteration in S1P‐receptor profile at night (parasympathetic predominance) and at daytime (sympathetic predominance). This is also in line with the previous study by Rossi et al., who reported that prevailing cardiac autonomic tone influences the heart rate decrease after the first dose of fingolimod (Rossi et al. 2015).

In‐hospital follow‐up lasting at least 6 h, as required in the drug label, might have had an effect on the circadian pattern of cardiac autonomic regulation at the day of fingolimod initiation. However, the possibility of physical (in)activity during in‐hospital follow‐up as a confounding factor remains low, because the night to day ratio of SDNN and TP, as well as the parameters of HRV reflecting mainly sympathetic cardiac regulation, remained unchanged between baseline and the day of fingolimod initiation.

## Conclusion

Physiological connection between circadian pattern of RR‐interval and overall HRV as well as cardiac parasympathetic regulation becomes uncoupled during continuous fingolimod treatment. In addition, fingolimod shifts circadian balance of cardiac autonomic regulation toward greater daytime enhancement. Accordingly, altered S1P‐receptor profile influences the endogenous circadian pattern of cardiac autonomic regulation in RRMS patients.

## Conflict of Interests

SS has been the congress representative of Mikkeli Central Hospital sponsored by industry (BiogenIdec, Boehringer Ingelheim, Genzyme, GlaxoSmithKline, Novartis, OrionPharma, Sanofi, TEVA) and has been a speaker sponsored by industry (BiogenIdec, Merck, Novartis, TEVA). TPL has received a research grant from the Finnish Foundation for Cardiovascular Research. TML: None. PH has been the congress representative of Kuopio University Hospital sponsored by industry (BiogenIdec, Genzyme, TEVA). JH has received research grants from the Finnish Foundation for Cardiovascular Research and the European Union Seventh Framework Programme and has been a speaker sponsored by industry (Amgen, AstraZeneca, Biotronic, Cardiome, MSD).

## Supporting information




**Table S1.** Heart rate variability during night and day before fingolimod initiation (B), at the day of fingolimod initiation (1D) and after 3 months of fingolimod treatment (3M). Absolute *P*‐values are presented in green.

**Table S2.** Night to day ratio for different heart rate variability measures before (B), at the day of fingolimod initiation (1D) and after 3 months of fingolimod treatment (3M). Absolute *P*‐values are presented in this supplementary Table.
