# Supplementary material for: Modulation of sphingosine receptors influences circadian pattern of cardiac autonomic regulation
Source: Physiol Rep. 2016 Sep 13;4(17):e12870. doi: 10.14814/phy2.12870 (PMC5027338; doi:10.14814/phy2.12870)
Supplement: Supplementary file 1 — Table S1. Heart rate variability during night and day before fingolimod initiation (B), at the day of fingolimod initiation (1D) and after 3 months of fingolimod treatment (3M). Absolute P‐values are presented in green. [file PHY2-4-12870-s001.docx]

Table S1. Heart rate variability during night and day before fingolimod initiation (B), at the day of fingolimod initiation (1D) and after three months of fingolimod treatment (3M). Absolute P-values are presented in green.

|  |  | Night | Day | *P-value* |
| --- | --- | --- | --- | --- |
| SDNN (ms) | B | 83 ± 28 | 89 ± 29 | *0.372* |
|  | 1D | 93 ± 28 | 103 ± 29 | *0.117* |
|  | 3M | 67 ± 23 | 74 ± 20 | *0.067* |
|  | *B vs 1D* | *0.009* | *0.000* |  |
|  | *B vs 3M* | *0.000* | *0.000* |  |
|  |  |  |  |  |
| pNN50 (%) | B | 16 ± 13 | 9.2 ± 8.3 | *0.001* |
|  | 1D | 23 ± 15 | 16 ± 11 | *0.002* |
|  | 3M | 10 ± 13 | 4.7 ± 4.8 | *0.027* |
|  | *B vs 1D* | *0.011* | *0.002* |  |
|  | *B vs 3M* | *0.002* | *0.013* |  |
|  |  |  |  |  |
| rMSSD (ms) | B | 43 ± 30 | 31 ± 19 | *0.001* |
|  | 1D | 51 ± 34 | 40 ± 20 | *0.026* |
|  | 3M | 29 ± 14 | 22 ± 8 | *0.008* |
|  | *B vs 1D* | *0.013* | *0.002* |  |
|  | *B vs 3M* | *0.000* | *0.003* |  |
|  |  |  |  |  |
| TP (ms x ms) | B | 5683 ± 3354 | 6402 ± 4128 | *0.348* |
|  | 1D | 6866 ± 3876 | 8858 ± 4814 | *0.074* |
|  | 3M | 3405 ± 1768 | 4635 ± 2588 | *0.130* |
|  | *B vs 1D* | *0.041* | *0.002* |  |
|  | *B vs 3M* | *0.000* | *0.004* |  |
|  |  |  |  |  |
| LFnu | B | 0.71 ± 0.16 | 0.78 ± 0.11 | *0.001* |
|  | 1D | 0.67 ± 0.15 | 0.72 ± 0.10 | *0.012* |
|  | 3M | 0.69 ± 0.15 | 0.77 ± 0.09 | *0.004* |
|  | *B vs 1D* | *0.006* | *0.000* |  |
|  | *B vs 3M* | *0.128* | *0.340* |  |
|  |  |  |  |  |
| HFnu | B | 0.28 ± 0.15 | 0.20 ± 0.10 | *0.001* |
|  | 1D | 0.31 ± 0.14 | 0.26 ± 0.09 | *0.004* |
|  | 3M | 0.30 ± 0.14 | 0.21 ± 0.08 | *0.001* |
|  | *B vs 1D* | *0.030* | *0.000* |  |
|  | *B vs 3M* | *0.170* | *0.093* |  |
|  |  |  |  |  |
| LF:HF-ratio | B | 4.37 ± 3.26 | 5.59 ± 2.87 | *0.009* |
|  | 1D | 3.38 ± 2.75 | 3.76 ± 2.49 | *0.119* |
|  | 3M | 3.49 ± 1.94 | 4.87 ± 248 | *0.004* |
|  | *B vs 1D* | *0.009* | *0.000* |  |
|  | *B vs 3M* | *0.072* | *0.049* |  |
