# Supplementary material for: Modulation of sphingosine receptors influences circadian pattern of cardiac autonomic regulation
Source: Physiol Rep. 2016 Sep 13;4(17):e12870. doi: 10.14814/phy2.12870 (PMC5027338; doi:10.14814/phy2.12870)
Supplement: Supplementary file 2 — Table S2. Night to day ratio for different heart rate variability measures before (B), at the day of fingolimod initiation (1D) and after 3 months of fingolimod treatment (3M). Absolute P‐values are presented in this supplementary Table. [file PHY2-4-12870-s002.docx]

Table S2. Night to day ratio for different heart rate variability measures before (B),
at the day of fingolimod initiation (1D) and after three months of fingolimod treatment (3M).
Absolute P-values are presented in this supplementary Table.

|  | B | 1D | 3M | P-value B vs 1D | P-value B vs 3M | P-value 1D vs 3M |
| --- | --- | --- | --- | --- | --- | --- |
| *Time Domain*  SDNN  pNN50  rMSSD  *Frequency Domain*  TP  LFnu  HFnu  LF:HF-ratio | 0.96 ± 0.29  2.92 ± 3.88  1.40 ± 0.55  0.97 ± 0.47  0.91 ± 0.16  1.38 ± 0.49  0.79 ± 0.40 | 0.94 ± 0.29  2.17 ± 2.57  1.26 ± 0.40  0.92 ± 0.53  0.93 ± 0.14  1.19 ± 0.29  0.87 ± 0.42 | 0.91 ± 0.24  2.94 ± 2.85  1.34 ± 0.45  0.82 ± 0.38  0.89 ± 0.17  1.47 ± 0.59  0.76 ± 0.42 | 0.596  0.048  0.047  0.555  0.315  0.018  0.323 | 0.085  0.522  0.141  0.023  0.567  0.745  0.728 | 0.637  0.594  0.253  0.196  0.080  0.018  0.108 |
